# Immunological Insights into the Life and Times of the Extinct Tasmanian Tiger (*Thylacinus cynocephalus*)

**DOI:** 10.1371/journal.pone.0144091

**Published:** 2015-12-14

**Authors:** Julie M. Old

**Affiliations:** Water and Wildlife Ecology, School of Science and Health, Hawkesbury, Western Sydney University, Locked Bag 1797, Penrith, NSW 2751, Australia; University of Illinois at Urbana-Champaign, UNITED STATES

## Abstract

The thylacine (*Thylacinus cynocephalus*) was Australia’s largest marsupial carnivore until its extinction within the last century. There remains considerable interest and debate regarding the biology of this species. Studies of thylacine biology are now limited to preserved specimens, and parts thereof, as well as written historical accounts of its biology. This study describes the development of the immune tissues of a pouch young thylacine, one of only eleven in existence, and the only specimen to be histologically sectioned. The appearance of the immune tissue of the developing pouch young thylacine is compared to the immune tissues of extant marsupials, providing insights into the immunity, biology and ecology of the extinct thylacine.

## Introduction

The thylacine (*Thylacinus cynocephalus*) is extinct [1], the last recorded specimen dying on the 7^th^ September, 1936 at Beaumaris Zoo in Hobart, Tasmania [2]. Nevertheless, reports of sightings in the Tasmanian wilderness continue to occur. Hunting and trapping associated with a significant bounty [3], disease [2, 4, 5] and potentially limited genetic diversity [6] led to its extinction.

The thylacine was the only extant member of the family Thylacinidae living into modern times. As evidenced by its fossil history, it was once more widely distributed on mainland Australia, New Guinea and Tasmania [2]. The thylacine was restricted to Tasmania at the time of initial European colonisation, although they were not regarded as a common species [4]. Thylacines were captured from most of Tasmania except the south, and south west ranges, with most caught on the central plateau according to bounty claims from 1888 to 1909, suggesting that their habitat consisted of open forest and woodland [2, 4]. It is likely that each thylacine had one range or territory, with reports by ‘tiger trappers’ at the time stating that the same animals were seen in the same areas [4].

*Thylacinus cynocephalus* [7] means ‘dog-headed pouched-dog’. The thylacine is therefore sometimes referred to as the ‘Tasmanian wolf’ due to the teeth, head and forequarters being canine-like in appearance, or ‘Tasmanian tiger’ due to the 13 to 19 distinctive black stripes distributed from the back of the body to a little past the base of the tail [4, 8]. The thylacine was a marsupial. Females had a backward facing pouch containing four teats and could raise a maximum of four pups, however usually only two or three young were reared to weaning [8].

Guiler [4] suggested that breeding occurred in December, based on reports at the time, and bounties were banned in December to avoid the breeding season. But bounty reports and payments also suggested that ‘pups’ could be found in the winter months and half-grown young a month later. More recent reports [9] have suggested that this hypothesis based on bounty data was likely incorrect due to hawking, the bulk submission of skins, and varying definitions of young in bounty reports. The recent review of newspaper articles, museum and zoological park records suggests that breeding occurred from April to September and pouch-dependent young were found from May to December [9].

Adult thylacines ranged in weight from 15–35kg [8]. The sexes were dimorphic, with males having wider necks and foreheads than the females [2]. Interestingly, male thylacines also had a backward facing pouch, however it contained the scrotal sac [10]. Longevity in captivity is likely to have been less than in the wild with one ‘cub’ (young at foot) held in the Beaumaris Zoo from 1924 to 1936, and another from the same litter dying one year previously [4]. In the wild thylacines may have lived for up to 12 to 14 years [4].

At present there are 14 known specimens of thylacine pouch young in existence [11]: four held at Charles University in Prague, five at the Tasmanian Museum and Art Gallery in Hobart, one at the Australian Museum in Sydney, and four at the Museum of Victoria in Melbourne. This paper describes the key immunological anatomy of one male pouch young thylacine, the only one in existence that has been sectioned, and provides insights into the immunology and biology of this extinct marsupial from a comparative perspective.

## Materials and Methods

### Specimen background and morphological description

The four pouch young currently held in the National Museum of Victoria were siblings collected with their mother from Launceston City Park on the 23^rd^ June, 1909 [11]. Boardman [12] described the external morphology of these specimens and gave their crown rump length measurements as approximately 75mm. Boardman [12] went on to describe the external morphology of all four pouch young as naked with the exception of some hair on the head and vibrissae. Some grey pigmentation was noted, mainly around the head, with the lips sealed laterally as described in other marsupial pouch young. The eyes were not yet open but the eyelashes were visible. The ears although not yet fully formed were present and pressed against the side of the head. Claws were present on the feet of all four pouch young and were black tipped.

### Estimation of pouch young age

The age of the pouch young specimens was estimated based on the following assumptions. Marsupials have an unchanging incremental daily increase in head length for the first 50 days of their pouch life, for example brushtail possums (*Trichosurus vulpecula*) [13], brush-tailed bettongs (*Bettongia penicillata*) [14], and Tasmanian devils (*Sarcophilus harrisii*) (L. Hughes, pers. comm.). The rate at which Tasmanian devil pouch young head lengths increase is 0.4mm/day for at least the first 50 days postpartum (L. Hughes, pers. comm.). If it is assumed that the thylacine also had an unchanging incremental daily increase in head length, and given that the largest adult Tasmanian devils weighed on average around 12.5kg [8], and thylacines averaged around 25kg [8], double the weight of a Tasmanian devil adult, we can presume the unchanging incremental increase in daily head length would be around twice that of the Tasmanian devil pouch young (0.8mm/day). Newborn Tasmanian devils have a head length of 2.8mm (L. Hughes, pers. comm.), and therefore we hypothesise that a newborn thylacine would have a head length measurement of around double that of the Tasmanian devil (5.6mm).

**Fig 1:**
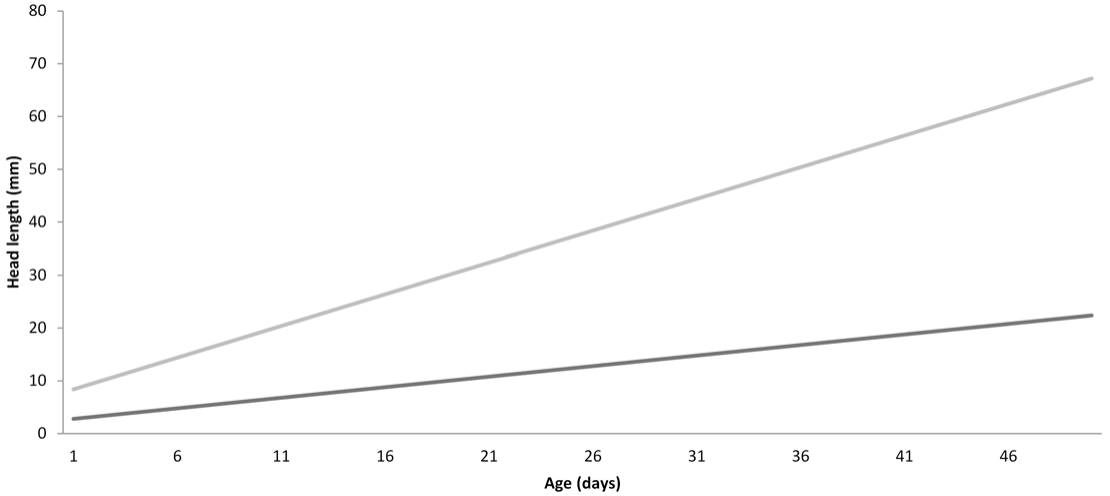
Predicted age of thylacine pouch young (grey) based on presumptive head length measurements (36mm), and compared to information on Tasmanian devils provided by L. Hughes (black).

Although we are unable to measure the head length of the sectioned thylacine pouch young, the head lengths were provided in Sleightholme et al. [11] for the three pouch young siblings of the sectioned pouch young. The mean head length of the three intact thylacine siblings was determined, and their age graphically extrapolated (Fig 1).

### Histology

One male pouch young (C5754), having been preserved in pure alcohol [11] was decalcified in nitric acid and processed before sectioning (10μm) for microscopy in 1994 by M. Klima. Sections were stained with either azan, or haematoxylin and eosin.

The histological slides from this specimen (loaned from the National Museum of Victoria and publicly available) were examined using an Olympus CX31 microscope (Olympus Corporation, Tokyo, Japan) and photomicrographs taken using an Olympus DP71 camera. Specifically, the thymus, bone marrow, spleen, gastrointestinal tract, liver, lungs and kidneys were examined. The thymus and bone marrow were examined as they are primary lymphoid tissues, whilst the spleen is a secondary lymphoid tissue along with the gastrointestinal tract and lungs. The gastrointestinal tract and lungs are potential sites for mucosal-associated lymphoid tissues, specifically, gut-associated and bronchus-associated lymphoid tissues, respectively. The liver was investigated because it is a primary site of haematopoiesis shortly after birth in marsupials (reviewed in [15, 16]). One further non-immune tissue was included, the kidneys, due to the uniqueness of the specimen.

## Results

The thylacine pouch young was processed for histology, sectioned and stained in 1994, having been stored in pure alcohol for nearly a century. The decades of long term storage in preservative, time since sectioning and staining had impacted the quality of the slides. It was difficult to discern specific cell types when viewed using high power magnification, with some sections showing evidence of other processing artifacts and damage, presumably also due to long term storage and age of the tissue generally. Despite some damage occurring to the sections and slides, most tissues could be described in some detail.

Although it was not possible to estimate the head length of this thylacine pouch young specimen (as it was already sectioned), the mean head length of its three siblings was 36mm [11]. The age of the pouch young thylacine was extrapolated from the graph and determined to be around 39 days postpartum.

### Thymus

**Fig 2:**
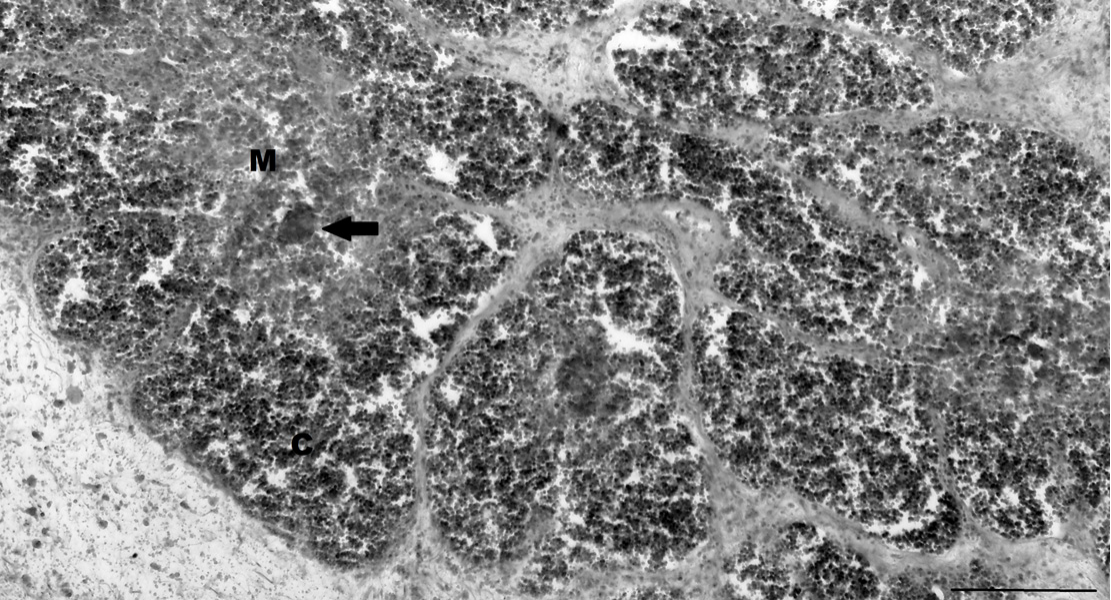
Pouch young thylacine thymus with well-defined lobules. Cortical regions (C) of the lobules contain densely packed lymphocytes whilst the medulla (M) is less densely packed and contains rare Hassall’s corpuscles (arrow). Scale bar 200μm.

Only a thoracic thymus was identified in the thorax section of the pouch young thylacine, with no cervical thymus located. The lobes and lobules of the thymus were separated by connective tissue septa (Fig 2). These septa were visible penetrating the cortical regions and ending at the cortico-medullary junctions. There were distinct areas of cortex and medulla with the cortex characteristically containing many more tightly packed lymphocytes than the medullary regions (Fig 2).

The early and mature lymphocytes of the thymus were uniform in appearance and size. Rare Hassall’s corpuscles were observed in the medullary regions of the thymus tissue sections (Fig 2).

### Bone marrow

Bone marrow was present in the long bones, the ribs and bones of the axial skeleton. Islands of haematopoietic areas were observed in the marrow spaces. These appeared to be mainly erythroblastic, although it was difficult to discern many of the cells.

**Fig 3:**
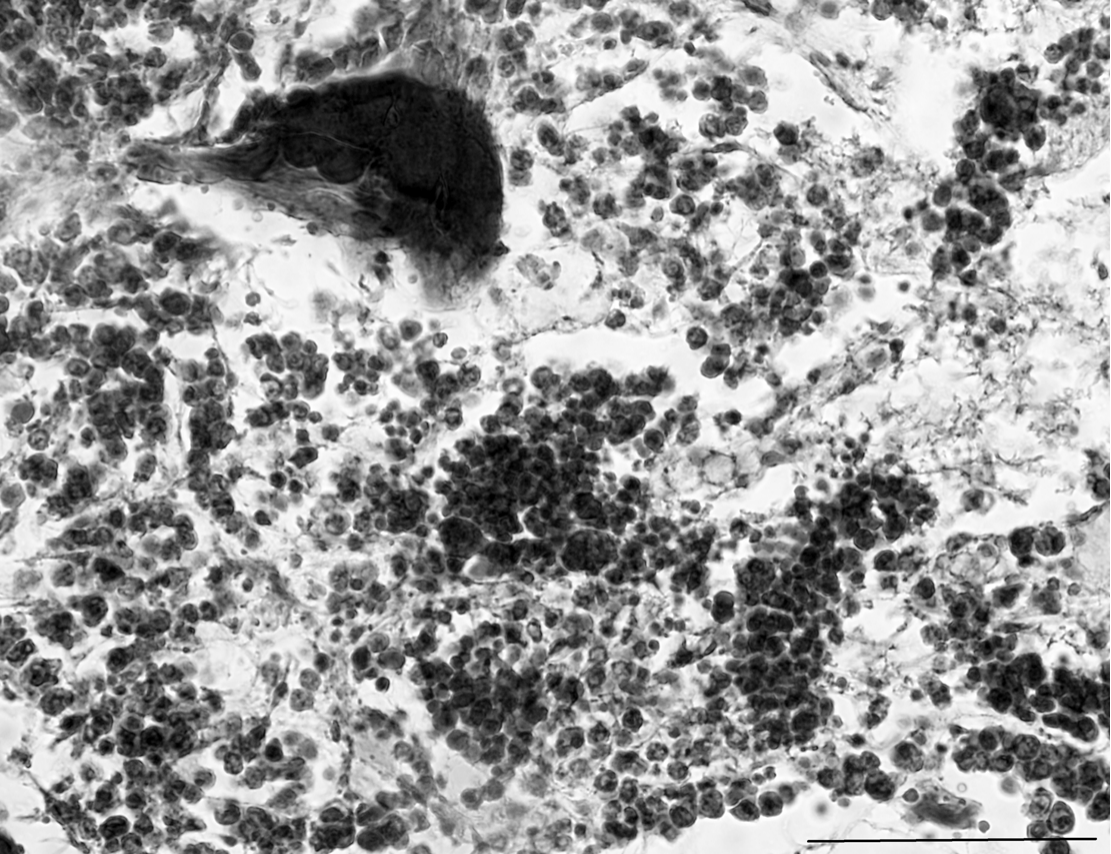
High magnification photomicrograph of pouch young thylacine bone marrow showing various cell types scattered throughout the medullary region. Scale bar 100μm.

Some sections of bone marrow were beginning to involute, with adipocytes present, despite many areas of haematopoiesis still evident (Fig 3). Other sections of bone marrow had no lipid cell infiltration. Occasional megakaryocytes were observed (Fig 3).

**Fig 4:**
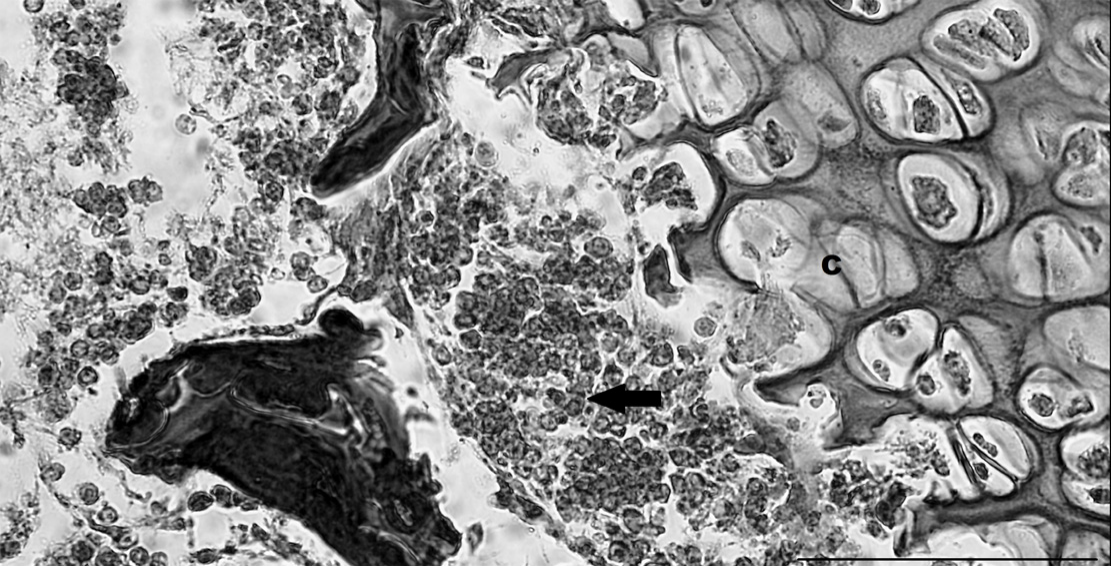
Pouch young thylacine long bone with rows of proliferating chondrocytes and chondrocytes (C) in lacunae. Islands of haematopoiesis (arrow) are evident in the medulla of the bone as well as some adipocytes. Scale bar 200μm.

The bones had some calcification occurring and some compact bone was visible. Some sections showed active growth of the bones and developing trabeculae were present. At a higher magnification, maturing cartilage cells were observed towards the bone epiphysis (Fig 4). Chondrocytes in lacunae and obvious rows of chrondrocytes proliferating were visible. Some chondrocytes were hypertrophied and the calcified matrix was forming.

The bone collar was composed of compact bone, and the trabeculae were visible. The periosteum had shrunk from the bone collar presumably due to the age and length of time the tissue had been in preservative solution, and tissue processing artifacts.

### Liver

**Fig 5:**
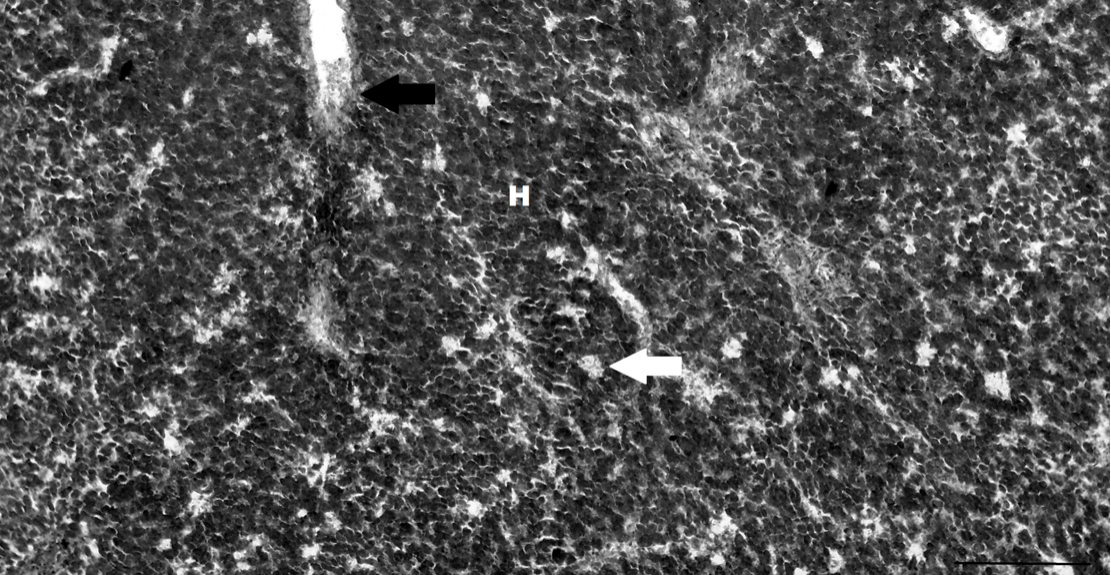
The liver of pouch young thylacine appears mature. Hepatocytes (H) and sinusoids are clearly observed radiating out from the central veins (white arrow), larger blood vessels are apparent (black arrow). No haematopoiesis is observed. Scale bar 200μm.

The liver had a characteristically mature appearance. There were hepatocytes, central and portal veins, and hepatic arteries visible (Fig 5), but no haematopoiesis. Sinusoids were evident between hepatocytes.

### Spleen

**Fig 6:**
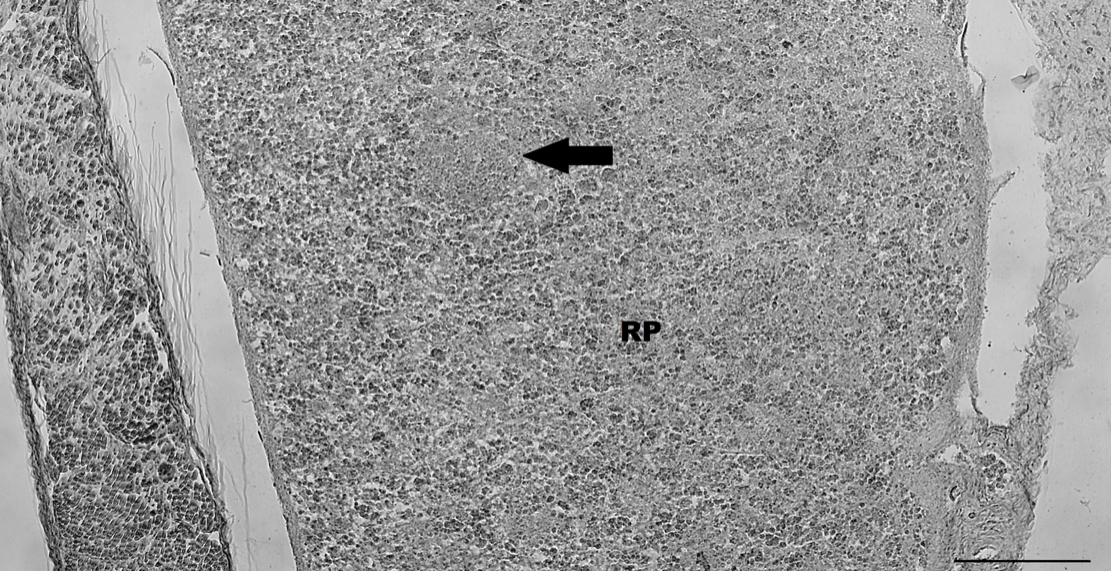
Pouch young thylacine spleen with some white and red pulp (RP) differentiation. White pulp areas contain more tightly packed cells and include one area that is likely an early follicle (arrow). Red pulp areas are less densely packed and contain areas with more darkly stained cells, presumably of the erythrocyte lineage. No mature follicles or germinal centres are apparent. Scale bar 200μm.

Due to the pouch young being sectioned transversely, only very thin sections of spleen could be viewed. The spleen was relatively uniform in appearance with some areas of white pulp beginning to appear (Fig 6). These areas contained one cell type that was uniform in size, appearance and staining pattern. One white pulp area was round and appeared to be a very early follicle. There was no marginal zone separating any of the white pulp areas from the surrounding red pulp. No mature follicles or germinal centres were present, and there were only very rare blood vessels seen. The majority of the red pulp was relatively uniform in appearance, but differing from white pulp areas, with a mix of different sized and differently stained cells present. No trabeculae were observed but a thin connective tissue capsule was present.

### Mucosal-associated lymphoid tissues

No mucosal associated lymphoid tissue was evident. Specifically, neither bronchus-associated lymphoid tissue in the lungs, nor gut-associated lymphoid tissues in the gastrointestinal tract were evident.

**Fig 7:**
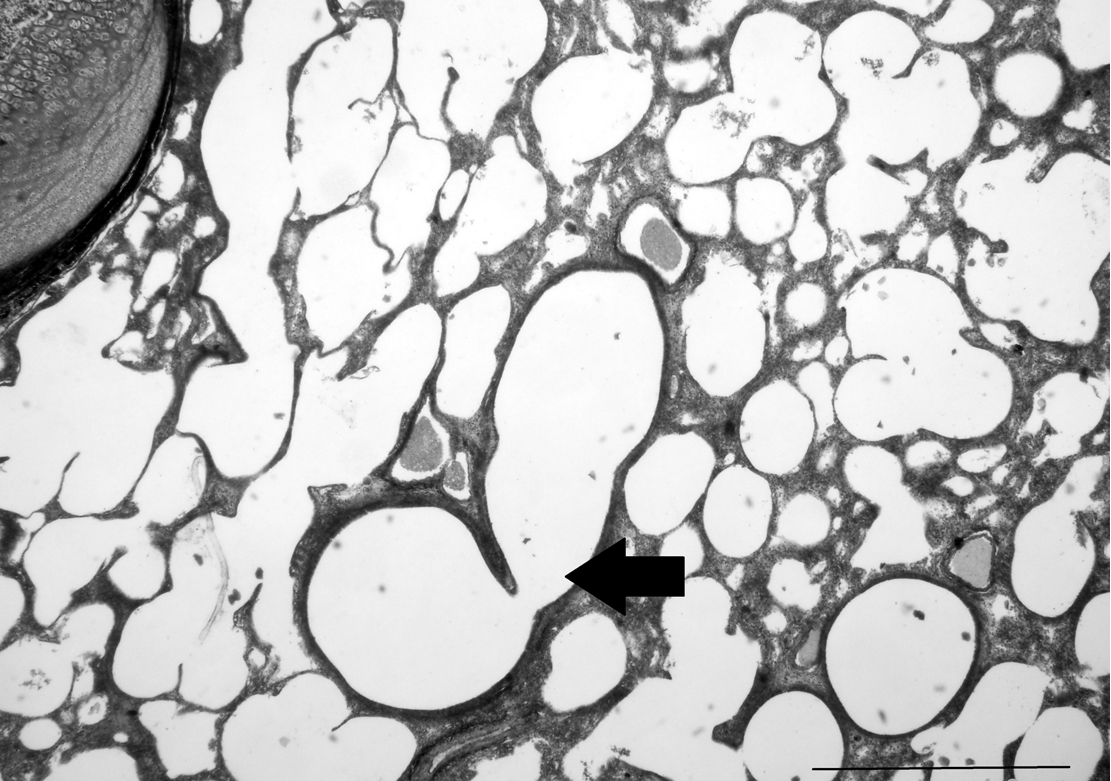
Lung of the pouch young thylacine with a range of different sized saccules evident throughout. Some blood vessels are seen within the parenchyma of the lung tissue. One large bronchus is indicated with an arrow. Epithelial cells are evident but there are no obvious lymphoid tissue accumulations. Scale bar 1μm.

The lungs were composed of large sacs, with some smaller saccules present at the extremities of the tissue (Fig 7). Small and large septa transversed the lung parenchyma, and the saccules varied in size. Secondary septa were sprouting out from the primary septa. Large blood vessels and bronchi could be seen. No lymphoid tissue accumulations or bronchial associated-cartilage was visible.

**Fig 8:**
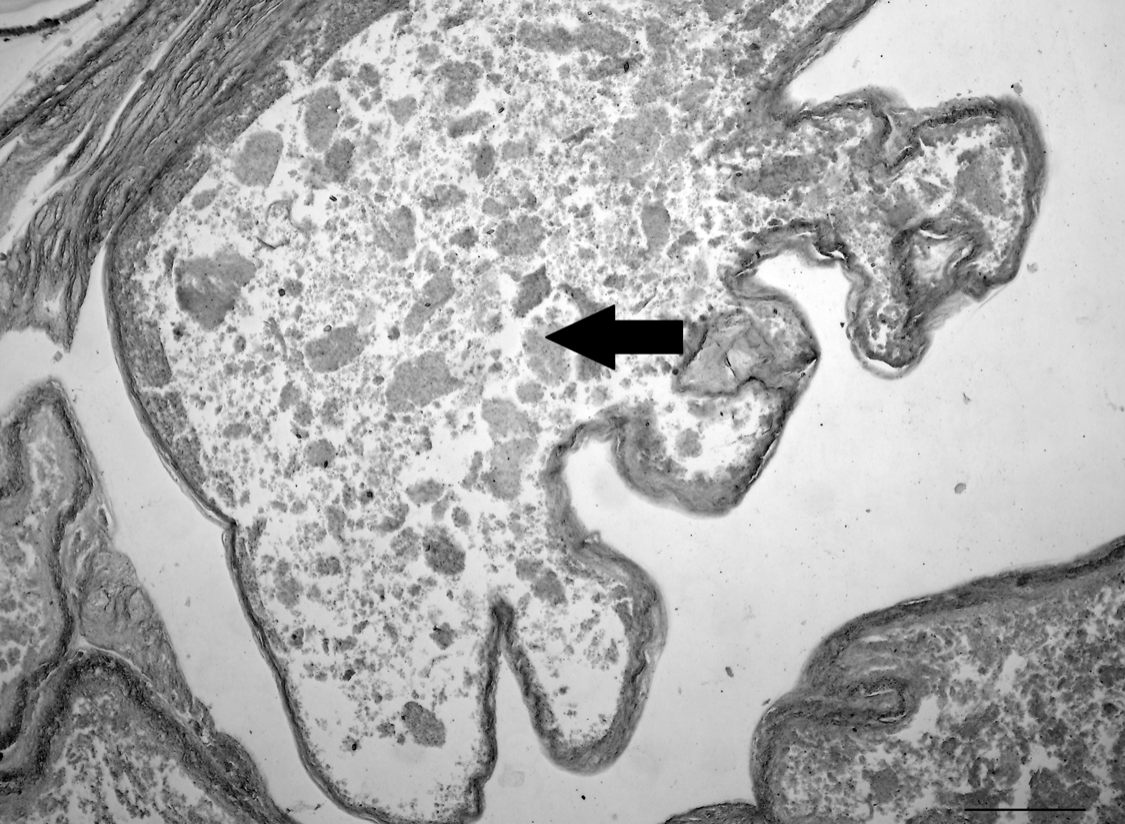
Photomicrograph showing thylacine pouch young gut. Note the lack of histological maturity of the gastrointestinal tract and little differentiation. The gut contents are likely milk (arrow). Scale bar 100μm.

The gastrointestinal tract itself was essentially a thin-walled sac. There were no villi, or intestinal glands and the mucosal and submucosal layers had not yet formed (Fig 8).

### Kidney

**Fig 9:**
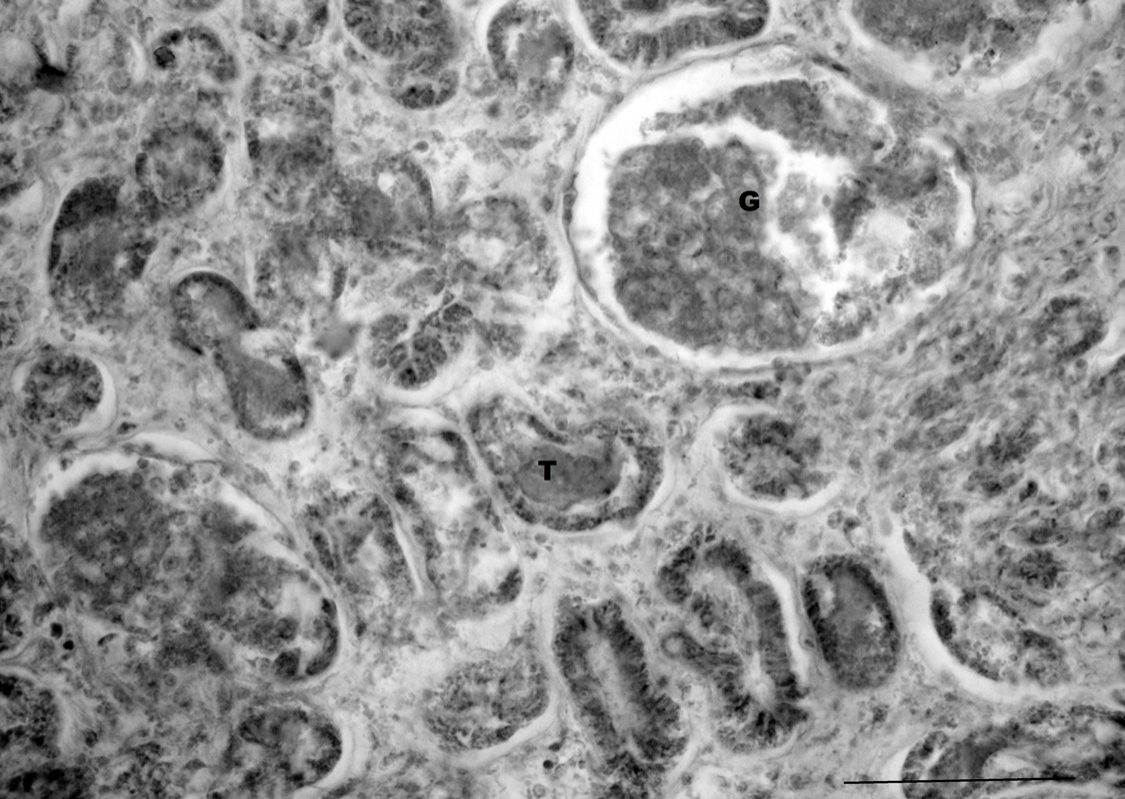
Photomicrograph of the pouch young thylacine kidney. Note the glomeruli (G) and tubules (T). Scale bar 100μm.

Glomeruli or renal corpuscles were visible with areas of white capillary loops and podocysts (Fig 9). Each glomerulus was surrounded by a urinary space and then a Bowman’s capsule. Large areas of the kidney were taken up by renal tubules. Distal convoluted tubules were visible and lacked microvilli. Proximal convoluted tubules with simple cuboidal epithelium and microvilli were evident. Large cleared areas were observed and identified as renal veins.

## Discussion

As one of only a few pouch young thylacines in existence, and the only one to have been serially sectioned, this specimen provided invaluable histological descriptions of the immune tissues of an extinct marsupial. It allowed a comparison of these tissues to those of some extant marsupials and provided insights into the biology of this extinct marsupial, albeit relying on a limited sample size.

The age of the pouch young in this study is unknown. Le Souef & Burrell [17] suggest that thylacines carried their young in the pouch for around three months, or eleven weeks, spending around one month in and out of the pouch, and by four months had permanently left the pouch. Many authors have questioned this timing (for example [4]) and it has been suggested that it is likely that the development of pouch young thylacines are more likely to follow a similar timeline to that of the Tasmanian devil, but others have stated that they have confidence in LeSouef & Burrell [17], and that this timeline is similar to the spotted-tailed quoll (*Dasyurus maculatus*) [2]. However there are currently no publicly available growth charts for most Dasyurid species, or the more closely-related numbat (*Myrmecobius fasciatus*), and generally a paucity of available literature on many of these key milestones for most marsupials. Only one study, for example, has described the developmental appearance of Tasmanian devil pouch young in captive bred animals of known age, but it was limited to one litter [18].

Boardman [12] provided an external morphological description of the thylacine pouch young, and Moeller provided an estimate of the age (one month) of the pouch young based on x-ray analysis of the skull and dentition [11]. In addition, the small dasyurid marsupial, the red-tailed phascogale (*Phascogale calura*), has claws present on all toes by day 30, fine fur appearing by around 34 days postpartum, and the young first detaching from the teat at day 42 postpartum [19], whilst Tasmanian devil pouch young have their first set of vibrissae erupt on day 33 after birth, and the young completely detach from the teat by 141 days [18]. Therefore based on the external morphological features of the thylacine pouch young, compared to that of extant marsupial pouch young, and based on the descriptions of Le Souef & Burrell [17], the pouch young specimen in this study was likely less than three months of age and nearing the time of first teat detachment. In addition, based on information and data provided by Hughes for the Tasmanian devil, the estimated age of the pouch young thylacines was extrapolated to be around 39 days postpartum, based on the mean head length measurement of its siblings of 36mm.

### Thymus

In contrast to the specimen examined in this study, Johnstone [20] described a mature thylacine (head length 21cm) thymus as standard in appearance when compared to all other vertebrates, having largely been infiltrated by adipose tissue with some strands of thymic cells remaining when examined histologically. To date this is the only description of thylacine primary immune tissue.

In this study of the pouch young thylacine only a thoracic thymus was observed. The lack of a second cervical thymus in the pouch young thylacine is similar to the findings in all other polyprotodont marsupials studied to date [21] and further supports the findings of Johnstone [20].

In marsupials the thymus is the first lymphoid tissue to mature (reviewed in [15, 16]). Previous studies have suggested a correlation between the presence of Hassall’s corpuscles, thymus maturity, and an ability to produce antibodies in marsupials [22, 23, 24]. Hassall’s corpuscles were observed in the pouch young thylacine thymus in this study suggesting it was mature. The young thylacine was therefore also likely to have been starting to produce antibodies of its own, and perhaps able to mount some of its own specific immune defence.

In marsupials like other vertebrates, lymphocytes differentiate in the cortical regions of the thymus and then move to the medullary regions. In marsupials this has been demonstrated with the use of a cross-species reactive antibody to the CD3ε chain developed by Jones et al. [25], and used in a range of marsupials (for example, [26, 27, 28, 29]). Specifically in the developing thymus mature T-cells (as defined by the positive staining of cells using the CD3ε marker) are distributed in the medulla. Unfortunately, no additional sections are available for antibody staining; however histological examination revealed well-defined areas of cortex and medulla, and it is likely that the thylacine thymus functions in a similar manner to that of other marsupials and vertebrates.

### Bone marrow

Only mature cells in the bone marrow could be identified with confidence. Ideally, fresh bone marrow samples and cross-species reactive antibodies would allow for less mature cells to be identified; however these options are currently not possible (for reviews on antibodies that cross-react with marsupial immune tissues see [15, 30]. Further the length and time the specimen was preserved are also likely to have affected the integrity of the specimen, in addition to decalcification, making any possible immunohistochemistry investigations problematic, even if all of the slides had not been stained histologically.

Comparatively little is known about bone marrow in marsupials, with only four species being investigated to date [22, 31, 32, 33]. In the species examined all had some bone marrow evident within two weeks of birth. Adipocytes infiltration started around 50 days postpartum in the stripe-faced dunnart (*Sminthopsis macroura*) [33], suggesting that the thylacine bone marrow was at least developmentally as old as a bone marrow sample from a 50 day postpartum stripe-faced dunnart.

Given the relative maturity of the bone marrow in the pouch young thylacine specimen, it was not unlikely to see a lack of haematopoiesis occurring in the liver. Marsupials are born with a haematopoietic liver rich in erythroblasts and to a lesser extent some granulocytopoiesis occurring [22, 31, 33, 34, 35]. Throughout pouch life, the liver rapidly matures and haematopoiesis ceases, with the bone marrow taking over the haematopoietic role (reviewed [15, 16]). Given that no haematopoiesis was present in the thylacine pouch young liver it suggests that in terms of development it was at least at the same developmental stage as the liver in a postpartum 120 day tammar wallaby (*Macropus eugenii*) [32], day 60 bandicoot (*Isodoon macrourus*) [35] and day 50 dunnart [33].

### Spleen

Although not fully developed the spleen was starting to mature, as there were large areas of red pulp, and some white pulp observed. Hartwig & Hartwig [36] suggested the relative volumes of red and white pulp, and trabeculae, can vary considerably depending on contraction or dilation of the spleen. The adult Virginian opossum (*Didelphis virginiana*) spleen, for example, lacked sinusoids, and trabeculae arteries were rare when described by Hayes [37].

The mature spleen is both an important site of immune defence, and storage site for the rapid release of erythrocytes. Hartwig & Hartwig [36] described the spleens of several mammalian species and provided examples in which the spleen anatomy can provide insights into the evolution of a species. Mammals with large blood vessels, a capsule and trabeculae within the spleen are likely to be able to rapidly release erythrocytes due to the higher levels of smooth muscle, whereas mammals with fewer blood vessels and higher levels of lymphoid tissue are less likely to use it for erythrocyte storage and utilise their spleen mainly for defence. Unfortunately, the spleen of the pouch young in this study is not yet developed enough to provide insights into the potential of the spleen to describe further aspects of the biology, such as the need to quickly supply erythrocytes to the muscle to allow prey to be chased down quickly, versus the ambush predator hypothesis. However, based on the anatomical structure of the jaw [38], teeth and axial skeleton [39] thylacines have been proposed to be hypercarnivores that likely preyed on species smaller than themselves, mainly by ambush and stealth, rather than pursuit predation. Paddle [2] reviewed the literature and personal accounts of the species and found that thylacines preferred fresh prey and that they were not known to eat carrion, only visiting a kill site once. Paddle [2] likewise found little evidence that thylacines preyed on poultry or livestock; the main reason the thylacine bounty was introduced. If juvenile or adult spleen sections became available, they may provide further support to the assertion that thylacines were ambush predators, which would not require the spleen to be a major erythrocyte storage site.

### Lymph nodes

Although no lymph nodes were observed in this study, it is highly likely that they were present in some form. When Johnstone [20] described the thymus in the mature thylacine, he stated that he found additional glandular structures and after histological examination found that they were lymph nodes. Unfortunately Johnstone [20] did not provide a further description of these cervical lymph nodes.

Lymph nodes have previously been difficult to locate in a range of marsupials [32, 40, 41, 42, 43]. The difficulty of finding lymph nodes in the thylacine in this study is likely a result of lymph nodes being missed during histological examination due to their small size, and potentially very under-developed appearance, or possibly being lost during serial sectioning of the specimen.

### Mucosal-associated lymphoid tissues

Although no lymphoid tissues were evident in the lungs or gut of the pouch young thylacine, this was not unexpected given the developmental stage of the specimen. In all marsupials to date the mucosal-associated lymphoid tissues are the last immune tissues to develop, and this also appears to be the case in the thylacine.

Whereas in peripheral lymphoid tissues such as the spleen and lymph nodes antigens are required for the tissues to fully mature, this is not always the case for bronchus-associated lymphoid tissues (BALT), with the appearance and frequency of BALT differing between and among species, and may or may not require antigen stimulation [44]. Bienenstock et al. [45] for example found that bronchus-associated lymphoid tissues can develop in germ free mice. In contrast, Emery & Dinsdale [46] found that lymphoreticular aggregates were not present at birth in humans but appeared at the end of the first week after birth, rapidly increasing in number, suggesting the need for exposure to antigen prior to their development. The occurrence of BALT in the lungs has been described in a few marsupials [41, 47, 48, 49, 50]. In the thylacine pouch young, the lack of BALT is similar to that described in the stripe-faced dunnart [41].

The thylacine pouch young lungs were more developed than those of newborn brushtail possums (*Trichosurus vulpecula*) or northern quolls (*Dasyurus hallucatus*) [51]. The newborn possum lung has been described as sac-like in appearance and the newborn quoll lung as more like two balloons, with each balloon-shaped lung lined and externally covered by epithelium [51].

The Virginian opossum does not have well developed alveoli until after 85 days postpartum when the pouch young starts to the leave the pouch intermittently [52]. Cooke & Alley [48] also found that brushtail possum lung did not resemble the adult lung until around 105 days postpartum (prior to pouch exit at 120 days [53]), and Gemmell [54] stated that the Northern brown bandicoot (*Isoodon macrourus*) lung was similar to the adult in appearance at 61 days after birth and corresponded to the time the young possum left the pouch. In addition, although Runciman et al. [55] described limited ages of developing lungs for the tammar wallaby, the pouch young thylacine investigated in this study had lungs with divisions in the terminal sacs, similar to that described in the tammar wallaby at 30 days postpartum. However, this stage of development continues in the wallaby until at least 70 days postpartum, with complete alveolisation not occurring until 180 days postpartum, and lung development continuing even after 400 days [55]. The thylacine lungs were also similar in appearance to the developing lungs of a quokka at 15 days postpartum age, with secondary septa sprouting out into the saccules from primary septa [56]. Based on the development of the lungs in the thylacine pouch young and comparing it to the observations of those in other marsupials, it provides additional support to suggest the pouch young was still pouch bound. Specifically, brushtail possum and Northern brown bandicoot lungs were not mature until around the time of pouch exit and were more mature in histological appearance compared to the thylacine lungs investigated in this study. The thylacine lungs were also similar in histological appearance to the pouch young tammar wallaby and quokka lungs at a time when they had not yet left the pouch [48, 52, 54, 55, 56].

Gemmell & Nelson [51] also suggested that there is no relationship between gestational length and maturation of the respiratory system in marsupials. However they do suggest that although gestational length does not appear to influence lung development at birth, newborn weight may, and this was supported in a further study on the development of lungs in the tammar wallaby [55]. Given that we do not know the weight of a newborn pouch young thylacine at birth, we would need to extrapolate it from extant marsupials. Tyndale-Biscoe [57] examined the relationship between maternal body weight and newborn body weight, and indicated that generally as maternal body weight increases, newborn body weight is also increased. As adult thylacines ranged in weight from 15–35kg (mean 25kg) and there was some sexual dimorphism between the sexes [8], based on the graph devised by Tyndale-Biscoe [57] we can assume a newborn thylacine might have weighed around 600–650mg. Therefore presumably the newborn thylacine had relatively more mature lungs than marsupials with a lower body weight at birth such as the brushtail possum and tammar wallaby.

The immature appearance of the gut and the external morphology of the pouch young provide further evidence that the pouch young was likely permanently attached to the teat and dependent on milk for sustenance, and maternal immunological protection, at the time of death. Milk in other marsupials has been found to contain a range of factors essential for growth, development, and immunological protection [53, 58, 59, 60, 61, 62], and unlike the milk produced by eutherian mammals, has different phases containing different components. In brushtail possums, for example, Adamski & Demmer [58] described three distinct phases of lactation. The first, referred to as the early lactation phase includes the period from birth to 80 days and is equivalent in terms of development to *in utero* in eutherians, hence could be considered as an ‘external gestation period’. During the second lactation or switch phase (80–120 days) the milk becomes more similar in composition to that of eutherian mammals. The third or late lactation phase occurs from 120–200 days postpartum in the possum (reviewed in [58]). The switch phase corresponds to when suckling becomes intermittent, the fur begins to grow and the eyes open in the brushtail possum [58]. In the pouch young thylacine in this study, Boardman [12] stated that the eyes were not yet open and the mouth was fused, but that the eyelashes were visible and although mostly naked had some hair on the head and vibrissae. These features suggest that the pouch young thylacine was likely still permanently attached to the teat and likely being supplied with early lactation phase milk, but is nearing the beginning of the switch phase, and first teat detachment.

### Kidneys

Kidneys were included in the description as they may provide some insights into the water conservation capacity of the thylacine, even though they are not immune tissues in mammals. Many Australian marsupials living in arid zones have thicker renal medullas (relative to overall kidney size) capable of producing urine of a higher concentration than those living in moister environments [63]. A thicker renal medulla incorporates longer renal tubules and hence allows greater water absorption and more concentrated urine. Given the relative lack of maturity of the pouch young, and the serial sectioning of the specimen, it is not possible to determine the relative thickness of the renal medulla as we are unable to determine the overall size of the kidney. Ideally an adult kidney would provide a better estimate of medullary thickness, as it would have reached maturity and presumably overall maximum size.

Despite the need for an adult kidney to accurately determine the relative renal thickness, and hence water conservation ability of the thylacine kidneys, the recorded distribution of the thylacine in Tasmania and New Guinea (currently moist habitats), suggests that thinner renal medullas are likely. However, thylacines were also previously distributed throughout mainland Australia [2] including fossil and non-fossil records from the Pleistocene and Holocene (a time of climate flux) [64]. The reason for the pre-modern era extinction of the thylacine from New Guinea and mainland Australia remains hotly debated (for example, [65]), and whether climate change and an inability to adapt may have contributed to their extinction in these areas remains debatable.

Unfortunately, the extinction of the thylacine precludes the collection and examination of additional samples for comparative purposes from the species. Nevertheless the description of the immune tissues in this one thylacine pouch young provides insights into the development of the pouch young thylacine immune system, and thylacine biology.
